# Radiological classification of the Heidelberg triangle and its application in laparoscopic pancreaticoduodenectomy for malignancies

**DOI:** 10.1186/s12957-023-03279-0

**Published:** 2024-01-03

**Authors:** Jiahao Chen, Abousalam Abdoulkader Ahmed, Jieqiong Ge, Zhiwei Cai, Xiao Hu, Xiaoyan Tang, Chunjing Li, Yunlong Pu, Chongyi Jiang

**Affiliations:** 1 Department of Hepato-Biliary-Pancreatic Surgery, Huadong Hospital Affiliated to Fudan University, Shanghai, 200040 China; 2 Shanghai Key Laboratory of Clinical Geriatric Medicine, Shanghai, China; 3 Department of Nursing, Huadong Hospital Affiliated to Fudan University, Shanghai, 200040 China

**Keywords:** Radiological classification, Heidelberg triangle, 3D reconstruction, Laparoscopy, Pancreaticoduodenectomy

## Abstract

**Background:**

The TRIANGLE operation benefits patients with pancreatic cancer; however, the Heidelberg triangle, where the operation occurs, contains vessels that can impact safety, especially in laparoscopic pancreaticoduodenectomy (LPD) with the TRIANGLE operation. This study aimed to identify Heidelberg triangle vessel types and their implications in pancreaticoduodenectomy (PD).

**Methods:**

Retrospective collection of radiographic data was performed from January 2017 to April 2023. Three-dimensional (3D) CT reconstructions were performed on patients. Vascular types in the Heidelberg triangle were classified based on named vessels crossing its interior. The impact of these types on surgical outcomes and complications in PD with the TRIANGLE operation was assessed.

**Results:**

Preoperative CT reconstruction was conducted on 184 pancreatic surgery patients. The findings revealed 99 patients (53.8%) with the type I Heidelberg triangle, lacking named vessels crossing the interior. Type II (*n* = 85, 46.2%), with named vessels crossing the interior, was identified. Among reconstructed patients who underwent PD with the TRIANGLE operation (*n* = 103), they were categorized as type I (*n* = 57) or type II (*n* = 46). The results showed that LPD patients with type II had significantly higher median intraoperative blood loss (300 mL vs. 200 mL, *P* = 0.030) and mean examined lymph nodes (17.2 ± 7.6 vs. 13.4 ± 5.2, *P* = 0.019) compared to those with type I. No significant differences were found in operative time or postoperative complications.

**Conclusion:**

The presence of named vessels crossing the interior of the Heidelberg triangle was associated with increased intraoperative bleeding during LPD combined with the TRIANGLE operation. Therefore, targeted preoperative planning is required before the operation, thus improving the safety of the TRIANGLE operation in minimally invasive surgery.

## Introduction

Pancreatoduodenectomy (PD) is the primary surgical procedure used to treat pancreatic head and periampullary tumors [1]. The safety of this procedure has been gradually improved over decades of refinement [2]. In line with the concept of minimally invasive surgery, laparoscopic pancreaticoduodenectomy (LPD) was first introduced by Gagner and Pomp in 1994 [3]. Over the past three decades, LPD has gained widespread acceptance, and various surgical techniques, such as “artery first,” have been proposed and applied to LPD [4, 5]. Surgeons who have mastered the necessary skills can now proficiently perform LPD after completing the learning curve [6].

In addition to ensuring the safety of the operation, achieving favorable margin status is also crucial, as patients with pancreatic cancer who have negative margins exhibit significantly longer overall survival than those with positive margins [7]. In 2017, the Pancreatic Center of the University of Heidelberg introduced the TRIANGLE operation. This procedure involves the thorough clearing of all blood vessels, lymph nodes, and nerve tissues in the triangular domain known as the Heidelberg triangle. The Heidelberg triangle is formed by the portal vein- superior mesenteric vein (PV-SMV), celiac axis (CA)-common hepatic artery (CHA), and superior mesenteric artery (SMA), and it involves skeletonization of these corresponding blood vessels. The TRIANGLE operation has been shown to improve the achievement of negative margins in patients with locally advanced pancreatic cancer who have undergone conversion therapy, potentially leading to extended survival time [8]. Understanding the anatomy of the Heidelberg triangle is of great importance for the safety of PD, especially in laparoscopic surgery [9–13]. The purpose of this study is to classify the types of vascular variation within the Heidelberg triangle region and to investigate their impact on the safety of LPD combined with the TRIANGLE operation, with the aim of improving surgical safety.

## Materials and methods

This retrospective cohort study was approved by the institutional review board of Huadong Hospital affiliated to Fudan University, and its protocol was declared on ClinicalTrials.gov (ID: NCT05703581) before statistical analysis on January 30, 2023. Informed consent was obtained from all patients after approval by the Ethics Committee of Huadong Hospital affiliated with Fudan University (No. 20170014). This study adhered to STROBE (Strengthening the Reporting of Observational Studies in Epidemiology) guidelines.

### Study design and patient selection

We retrospectively collected preoperative computed tomography (CT) images of patients who underwent pancreatic surgery at Huadong Hospital affiliated with Fudan University between January 2017 and April 2023. These images were used for three-dimensional reconstructions and subsequent categorization. Since our center initiated the TRIANGLE operation in June 2019, we gathered clinical data from patients who underwent PD combined with TRIANGLE operation from June 2019 to April 2023 for further analysis. All patients included in our data analysis presented with resectable tumors and did not undergo preoperative chemotherapy. The exclusion criteria were as follows: (1) patients who did not undergo PD and (2) patients who did not undergo the TRIANGLE operation. Based on whether named vessels crossed the interior of the Heidelberg triangle, the selected patients were divided into type I and type II, and their baseline levels, surgical and pathological results, and the incidence of complications were compared. All operations were performed by the same surgical team, which had surpassed the learning curve for both LPD and open pancreaticoduodenectomy (OPD) [14, 15].

### CT and 3D reconstruction

Preoperative contrast-enhanced CT images with a slice thickness ≤ 1.25 mm were retrospectively collected and imported into the Mimics 20.0 software (Materialise, Belgium) for 3D reconstruction. The methodology employed in this study involved the utilization of the dynamic region growing function to segment the specific region of interest within the mask. Subsequently, the blood vessels within the Heidelberg triangle were calculated, and a process of wrapping and smoothing was applied to create a comprehensive three-dimensional structural model of the blood vessels within the Heidelberg triangle. The arterial phase and portal venous phase images of each patient were reconstructed to generate stereolithography (STL) files, and the STL registration function was utilized to align and register the two-stage three-dimensional models. This process culminated in the acquisition of a complete and accurate vascular model of the Heidelberg triangle.

### Surgical procedure

Our institution performed LPD and OPD using a standard approach. The surgical technique involved the use of a 5-hole method. Once the peritoneal cavity was accessed, the same surgical steps were performed for both OPD and LPD. The patients who underwent the TRIANGLE operation also received standard lymphadenectomy as per the International Study Group of Pancreatic Surgery (ISGPS) consensus [16], in addition to undergoing Heidelberg triangle dissection during the PD operation. The dissection of the Heidelberg triangle entailed the meticulous removal of all vascular, lymphoid, and neural tissues within the triangular area enclosed by the PV-SMV, CA-CHA, and SMA. The three vessels enclosing the Heidelberg triangle were carefully skeletonized [8]. Following the removal of the specimens, we employed a modified version of Child’s method to accomplish intestinal reconstruction. This involved performing a pancreatojejunostomy based on the Blumgart anastomosis technique [17] with a duct-to-mucosa, end-to-side approach, and placing a pancreatic drainage tube. The biliary-enteric anastomosis was accomplished using a continuous suture technique, while gastrojejunostomy was achieved using interrupted 3–0 polypropylene monofilament sutures.

### Definitions and data collection

Baseline data, such as sex, age, body mass index (BMI), American Society of Anesthesiologists (ASA) score, main pancreatic duct size, carbohydrate antigen 19–9 (CA19-9), carcinoembryonic antigen (CEA), albumin, and serum bilirubin, were obtained electronically from the hospital laboratory information system. Pathologic data, including grading, number of examined lymph nodes (ELNs), rate of lymph node positivity and margin status, were retrieved from the pathology database. Postoperative complications, such as postoperative pancreatic fistula (POPF) [18], bile leakage [19], chyle leak [20], delayed gastric emptying (DGE) [21], and postpancreatectomy hemorrhage (PPH) [22], were classified based on the definitions provided by the ISGPS, including clinically relevant complications (grades B and C according to ISGPS). The approach our center adopts for estimating blood loss is based on the formula: (suction canister fluids count − abdominal irrigation fluids) + (operative gauze weight − dry gauze weight). This method is among the most commonly used in hepato-pancreato-biliary surgeries to estimate intraoperative blood loss [23]. For the sake of binary logistic analysis, “high intraoperative blood loss” was defined as twice the median value. This categorization method was not arbitrarily based solely on the percentile distribution of the intraoperative blood loss but allowed for a significant elevation in intraoperative blood loss above the median value (by a factor of two), regardless of the number of patients exhibiting high intraoperative blood loss in the study cohort [24].

### Statistical analysis

Statistical analysis was conducted to analyze the data. For continuous variables exhibiting a normal distribution, the mean value and standard deviation (SD) were reported and analyzed using Student’s *t*-test. Similarly, for continuous variables demonstrating a non-normal distribution, the median and interquartile range (IQR) were presented and analyzed using the Mann–Whitney *U*-test. Depending on the specific situation, the categorical variables were analyzed using the chi-square test or Fisher’s exact test. Risk factors for high intraoperative blood loss were assessed using univariate binary logistic regression. Variables with *P* < 0.05 in the univariate analysis were subsequently included in a multivariate binary logistic regression model (Method: Enter). All tests were conducted with a two-tailed approach, with a statistical significance set at 0.05. All statistical analyses were performed using SPSS V.26 software (IBM, USA).

## Results

### Vascular classification of the Heidelberg triangle

**Fig. 1:**
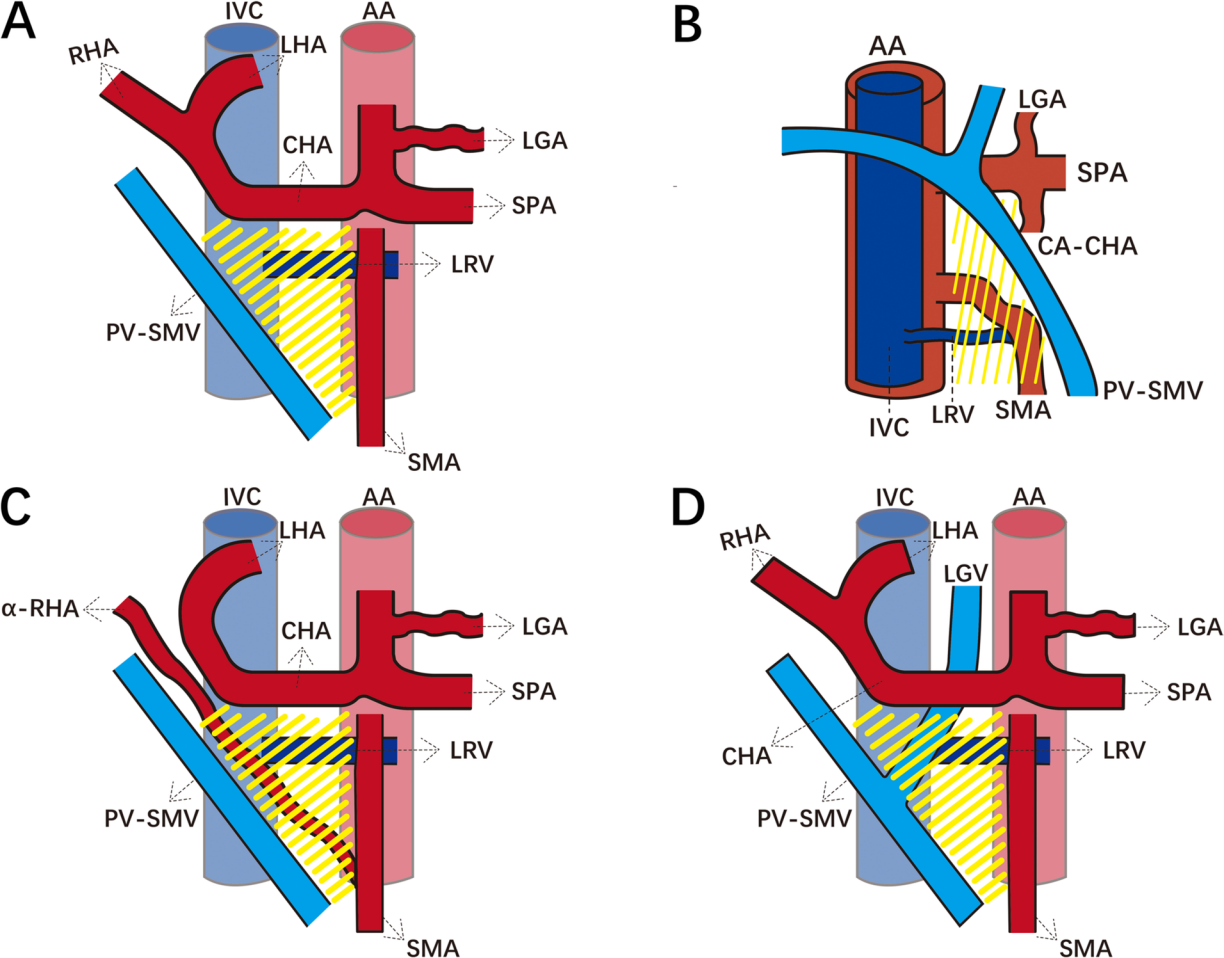
Schematic figures of the Heidelberg triangle classification. **A** Ventral view of the type I Heidelberg triangle. Yellow shading refers to the interior of the Heidelberg Triangle. **B** Lateral view of the type I Heidelberg triangle. The interior of the Heidelberg triangle, shown in yellow, is defined as the triangular plane formed by the posterior edge of the CA-CHA, SMA, and PV-SMV vessels extending to the plane of the inferior vena cava and left renal vein. **C** Ventral view of the type IIa Heidelberg triangle. **D** Ventral view of the type IIv Heidelberg triangle. Abbreviations: AA, abdominal aorta; CA, celiac axis; CHA, common hepatic artery; SMA, superior mesenteric artery; SPA, splenic artery; LGA, left gastric artery; α-RHA, variant right hepatic artery; LHA, left hepatic artery; RHA, right hepatic artery; LGV, left gastric vein; SMV, superior mesenteric vein; PV, portal vein; IVC, inferior vena cava; LRV, left renal vein

**Fig. 2:**
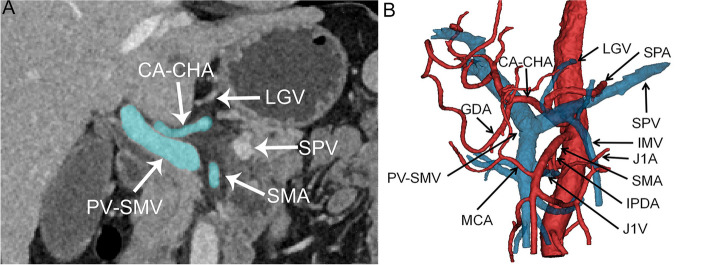
Coronal CT images and three-dimensional reconstructed images of the type I Heidelberg triangle. **A** Coronal CT images of the type I Heidelberg triangle, characterized by the absence of any named vessel crossing the interior of the Heidelberg triangle, accounted for 53.8% of reconstructed patients. The blue area indicates the boundary vessels of the Heidelberg triangle, and the white arrowhead points to vessels around the Heidelberg triangle. **B** The three-dimensional reconstructed image of the type I Heidelberg triangle is shown. The black arrowhead points to vessels around the Heidelberg triangle. Veins are depicted in blue, while arteries are represented in red. Abbreviations: CA, celiac axis; CHA, common hepatic artery; SMA, superior mesenteric artery; SPA, splenic artery; GDA, gastroduodenal artery; IPDA, inferior pancreaticoduodenal artery; J1A, first jejunal artery; MCA, middle colic artery; PV, portal vein; SMV, superior mesenteric vein; LGV, left gastric vein; SPV, splenic vein; IMV, inferior mesenteric vein; J1V, first jejunal vein

**Fig. 3:**
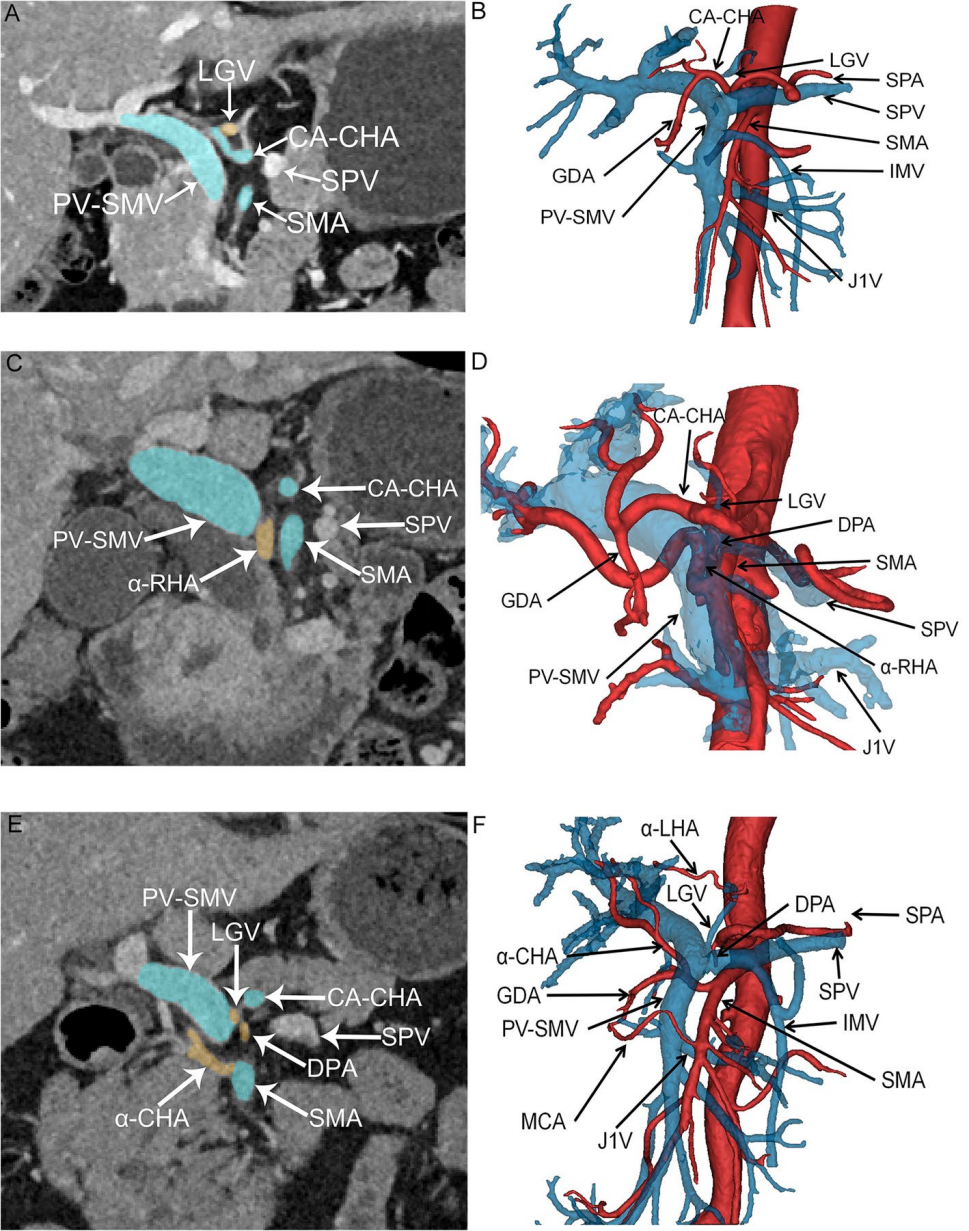
Coronal CT images and three-dimensional reconstructed images of the type II Heidelberg triangle. **A**, **C**, **E** Coronal CT images depicting the type II Heidelberg triangle, with named vessels crossing the interior of the Heidelberg triangle, accounting for 46.2% of reconstructed patients. The blue area represents the boundary vessels of the Heidelberg triangle, and the orange area indicates the vessels crossing through the interior of the Heidelberg triangle. The white arrowhead points to vessels around the Heidelberg triangle. **B**, **D**, **F** The three-dimensional reconstructed image of the type II Heidelberg triangle. The black arrowhead points to vessels around the Heidelberg triangle. Veins are depicted in blue, while arteries are represented in red. Abbreviations: CA, celiac axis; CHA, common hepatic artery; SMA, superior mesenteric artery; DPA, dorsal pancreatic artery; SPA, splenic artery; LGA, left gastric artery; GDA, gastroduodenal artery; IPDA, inferior pancreaticoduodenal artery; α-RHA, variant right hepatic artery; α-LHA, variant left hepatic artery; α-CHA, variant common hepatic artery; MCA, middle colic artery; J1A, first jejunal artery; LGV, left gastric vein; SMV, superior mesenteric vein; PV, portal vein; SPV, splenic vein; IMV, inferior mesenteric vein; J1V, first jejunal vein

We performed 3D reconstruction of preoperative CT scans from 184 patients who underwent pancreatic surgery. The front of the triangle was defined as the plane formed by the three boundary vessels of the triangle extending toward the posterior edge of the pancreas. The findings revealed that, in most cases, the blood vessels passed in front of the Heidelberg triangle and did not cross through the interior of the triangle. This vascular arrangement was observed from the cranial to caudal direction, including the left gastric vein (LGV), splenic artery (SPA), dorsal pancreatic artery (DPA) arising from the SPA, splenic vein (SPV), inferior mesenteric vein (IMV), inferior pancreaticoduodenal artery (IPDA), first jejunal artery (J1A), and middle colic artery (MCA). The interior of the Heidelberg triangle was defined as the triangular plane formed by the posterior edge of the CA-CHA, SMA, and PV-SMV vessels extending to the plane where the inferior vena cava and left renal vein are located. Normally, no named arteries or veins are observed in this area (Fig. 1). This vascular arrangement was categorized as type I, comprising 99 cases, which accounted for 53.8% of the reconstructed patients (Fig. 2). Given the challenging exposure of blood vessels within the triangle during surgery, our focus was on the classification of these vessels. Specifically, the LGV crossed the interior of the triangle and drained into the PV, accounting for 37% of the reconstructed patients. In 10 patients (5.4%), the DPA crossed the interior of the triangle, with 5 originating from the SMA and the other 5 from the CHA. A total of 24 cases (13.0%) of variant hepatic arteries were found to cross the interior of the triangle, 18 of which were variant right hepatic arteries (α-RHA) originating from the SMA, and the other 6 were variant common hepatic arteries (α-CHA) originating from the SMA. In addition, two patients (1.1%) had MCAs that crossed the interior of the triangle and originated from the CA. Patients with any named vessel crossing the interior of the triangle were classified as type II (*n* = 85, 46.2%). We further subdivided type II into type IIa (arterial type, with named arteries crossing the interior of the Heidelberg triangle) and type IIv (venous type, with named veins crossing the interior of the Heidelberg triangle but named arteries are absent) (Fig. 1C, D). Representative CT and 3D reconstructed images demonstrating type II are depicted in Fig. 3.

### Cohort characteristics

**Fig. 4:**
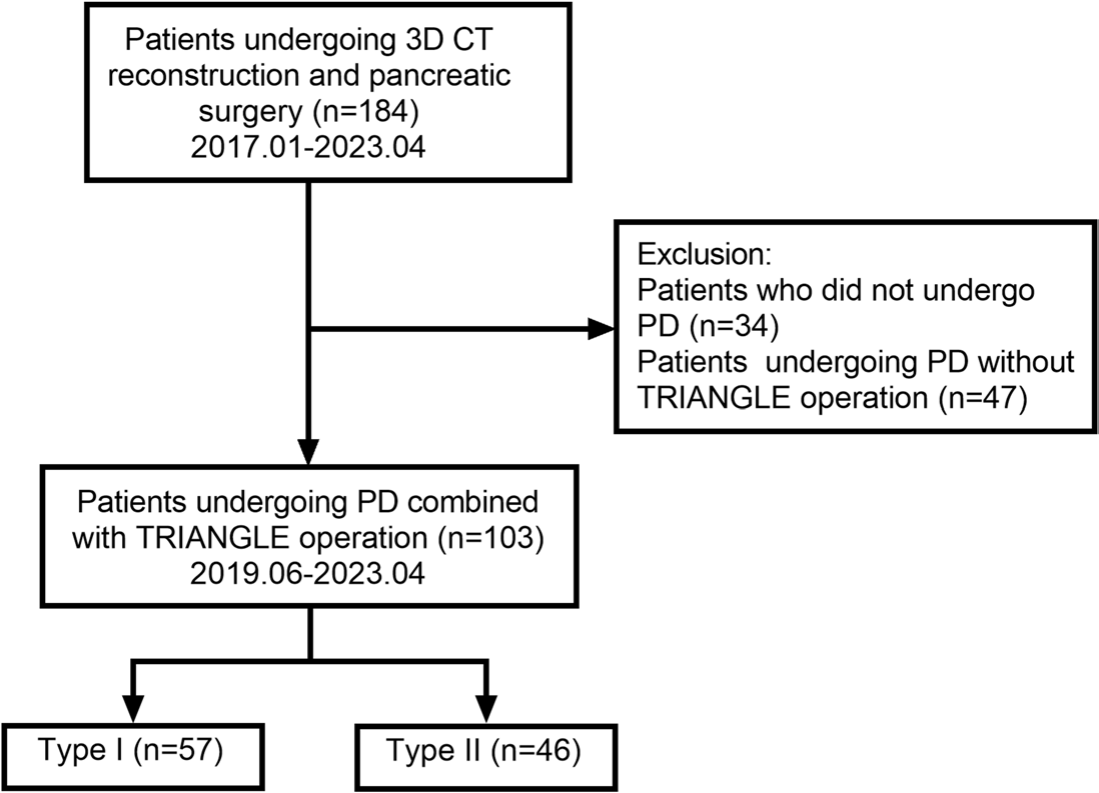
Flow chart of patient selection. Abbreviations: 3D, three-dimensional; CT, computed tomography; PD, pancreaticoduodenectomy

To verify the impact of the Heidelberg triangle vascular classification on PD combined with the TRIANGLE operation, we further screened patients who had undergone reconstruction. Out of the 184 patients who had undergone 3D reconstruction, we excluded 34 patients who had undergone surgical procedures other than PD, such as central pancreatectomy and distal pancreatectomy. Additionally, 47 patients who did not undergo the TRIANGLE operation were also excluded. Finally, our analysis included 103 patients who underwent PD combined with the TRIANGLE operation. All 103 patients included in our data analysis presented with resectable tumors and did not undergo preoperative chemotherapy. These patients were divided into type I (*n* = 57) and type II (*n* = 46) based on the presence of named vessels crossing through the interior of the Heidelberg triangle (Fig. 4). The results demonstrated that, aside from a difference in the proportion of LPD patients (75.4% vs. 54.4%, *P* = 0.025), there were no significant disparities between type I and type II patients in terms of basic demographics such as gender, age, body mass index (BMI), ASA score, and main pancreatic duct size. Similarly, serum markers including albumin, serum bilirubin, CA19-9, and CEA showed no notable variation between the two groups, indicating comparable baseline characteristics for both types (Table 1). To mitigate the potential influence of different LPD proportions on subsequent results, we conducted identical analyses separately for both LPD and OPD populations. The results indicated that, even after segregating the LPD and OPD groups for analysis, the baseline parameters between type I and type II patients remained balanced and comparable (Table 2).

**Table 1:** Baseline characteristics

Variables	Type I (*n* = 57)	Type II (*n* = 46)	*P* value
Sex			0.064
Male	23 (40.3)	27 (58.7)	
Female	34 (59.7)	19 (41.3)	
Age (years)^a^	67.3 ± 11.7	66.5 ± 9.0	0.695
BMI (kg/m^2^)^a^	22.57 ± 3.03	23.18 ± 3.19	0.320
Surgical type			0.025
LPD	43 (75.4)	25 (54.4)	
OPD	14 (24.6)	21 (45.6)	
ASA score			0.403
1	6 (10.5)	7 (15.2)	
2	43 (75.5)	36 (78.3)	
3	8 (14.0)	3 (6.5)	
Main pancreatic duct size (mm)			0.297
≤ 3	23 (40.4)	14 (30.4)	
> 3	34 (59.7)	32 (69.6)	
Albumin (g/L)^b^	41.5 (38.0, 44.0)	40.7 (36.3, 42.0)	0.150
Serum bilirubin (μmol/L)^b^	14.2 (9.0, 55.7)	36.1 (8.6, 183.7)	0.140
CA19-9 (IU/mL)	40 (15.5, 223.4)	75.7 (24.5, 149.0)	0.573
CEA (ng/mL)	2.5 (1.9, 4)	2.5 (1.9, 4.3)	0.974
Tumor size (mm)	25 (20, 35)	25 (20, 35)	0.520

Values in parenthesis are percentages unless indicated otherwise:

*Abbreviations*: *LPD* laparoscopic pancreaticoduodenectomy, *OPD* open pancreaticoduodenectomy, *SD* standard deviation, *IQR* interquartile range, *BMI* body mass index, *ASA* American Society of Anesthesiologists, *CA 19–9* carbohydrate antigen 19–9, *CEA* carcinoembryonic antigen

^a^ mean ± SD; ^b^ values are median (IQR)

**Table 2:** Baseline characteristics in LPD and OPD

Variables	LPD (*n* = 68)			OPD (*n* = 35)		
	Type I (*n* = 43)	Type II (*n* = 25)	*P* value	Type I (*n* = 14)	Type II (*n* = 21)	*P* value
Sex			0.149			0.305
Male	18 (41.9)	15 (60.0)		5 (35.7)	12 (57.1)	
Female	25 (58.1)	10 (40.0)		9 (64.3)	9 (42.9)	
Age (years)^a^	67.3 ± 12.1	69.0 ± 8.9	0.532	67.2 ± 10.8	63.4 ± 8.4	0.247
BMI (kg/m^2^)^a^	23.0 ± 3.0	22.1 ± 2.9	0.240	23.3 ± 2.6	24.4 ± 3.1	0.271
ASA score			0.490			1.000
1	4 (9.3)	4 (16.0)		2 (14.3)	3 (14.3)	
2	32 (74.4)	19 (76.0)		11 (78.6)	17 (81.0)	
3	7 (16.3)	2 (8.0)		1 (7.1)	1 (4.7)	
Main pancreatic duct size (mm)			0.398			1.000
≤ 3	20 (46.5)	9 (36.0)		3 (21.4)	5 (23.8)	
> 3	23 (53.5)	16 (64.0)		11 (78.6)	16 (76.2)	
Albumin (g/L)^b^	41.0 (35.7, 45.0)	39.5 (34.4, 42.0)	0.130	41.8 (40.0, 43.1)	41.0 (38.3, 42.0)	0.448
Serum bilirubin (μmol/L)^b^	15.5 (9.9, 56.5)	29.6 (11.0, 162.4)	0.182	12.2 (8.2, 24.5)	62.1 (8.4, 211.5)	0.419
CA19-9 (IU/mL)^b^	30.0 (13.3, 91.6)	69.0 (23.3,138.8)	0.294	56.7 (29.1, 620.0)	96.0 (28.0, 156.0)	0.429
CEA (ng/mL)^b^	2.5 (1.9, 3.4)	2.9 (1.9, 3.8)	0.558	3.7 (2.4, 7.8)	2.3 (2.0, 4.7)	0.288
Tumor size (mm)^b^	25.0 (20.0, 32.5)	25.0 (20.0, 30.0)	0.742	30.0 (30.0, 38.8)	30.0 (20.0, 35.0)	0.226

Values in parenthesis are percentages unless indicated otherwise:

*Abbreviations*: *LPD *laparoscopic pancreaticoduodenectomy, *OPD *open pancreaticoduodenectomy, *SD *standard deviation, *IQR *interquartile range, *BMI *body mass index, *ASA *American Society of Anesthesiologists, *CA 19–9 *carbohydrate antigen 19–9, *CEA *carcinoembryonic antigen

^a^ mean ± SD; ^b^ values are median (IQR)

### Perioperative outcomes for LPD and OPD

Perioperative indexes and complications of type I and type II were compared within the two surgical procedures. The results showed no significant difference in median operation time between type I and type II in LPD (370 min vs. 420 min, *P* = 0.102). Similarly, no significant difference in median operation time was observed between type I and type II in OPD (378 min vs. 330 min, *P* = 0.479). Notably, in LPD, the median intraoperative blood loss of type II was significantly higher than that of type I (300 mL vs. 200 mL, *P* = 0.030). However, in OPD, there was no significant difference in the median intraoperative blood loss between type I and type II (300 mL vs. 400 mL, *P* = 0.564), suggesting that type II significantly increased the intraoperative blood loss of LPD (Table 3). Additionally, there was no significant difference in intraoperative blood transfusion rate and LOS between type I and type II in both surgical procedures. The total complication rate, POPF (B/C), bile leakage (B/C), chyle leak (B/C), DGE (B/C), PPH (B/C), intra-abdominal infection, and the 90-day postoperative mortality rate did not significantly differ between the type I and type II subgroups of LPD and OPD (Table 3).

**Table 3:** Surgical and pathologic outcomes

Variables	LPD (*n* = 68)			OPD (*n* = 35)		
	Type I (*n* = 43)	Type II (*n* = 25)	*P* value	Type I (*n* = 14)	Type II (*n* = 21)	*P* value
Operative time (min)^a^	370 (340, 437)	420 (365, 474)	0.102	378 (325, 420)	330 (292, 420)	0.479
Intraoperative blood loss (mL)^a^	200 (100, 300)	300 (200, 450)	0.030	300 (163, 475)	400 (200, 650)	0.564
Intraoperative transfusion			0.744			1.000
Yes	4 (9.3)	1 (4.0)		4 (28.6)	5 (23.8)	
No	39 (90.7)	24 (96.0)		10 (71.4)	16 (76.2)	
LOS (days)^a^	14 (12, 18)	14 (11, 22)	0.636	14 (12, 29)	15 (12, 20)	0.973
Surgical morbidity	16 (37.2)	13 (52.0)	0.234	7 (50.0)	7 (33.3)	0.483
POPF (B/C)	9 (20.9)	5 (20.0)	0.927	4 (28.6)	4 (19.1)	0.685
Bile leakage (B/C)	4 (9.3)	1 (4.0)	0.744	0 (0.0)	1 (4.8)	1.000
Chyle leak (B/C)	1 (2.3)	2 (8.0)	0.627	0 (0.0)	1 (4.8)	1.000
DGE (B/C)	0 (0.0)	2 (8.0)	0.132	1 (7.1)	2 (9.5)	1.000
Intra-abdominal infection	12 (27.9)	9 (36.0)	0.486	3 (21.4)	6 (28.6)	0.712
PPH (B/C)	2 (4.7)	3 (12.0)	0.524	1 (7.1)	1 (4.8)	1.000
Reoperation	1 (2.3)	1 (4.0)	1.000	0 (0.0)	0 (0.0)	/
90-day mortality	2 (4.7)	1 (4.0)	1.000	0 (0.0)	0 (0.0)	/
Histology			0.589			0.847
Pancreatic head cancer	24 (55.8)	17 (68.0)		10 (71.4)	16 (76.2)	
Ampullary carcinoma	13 (30.2)	5 (20.0)		3 (21.4)	3 (14.3)	
Distal cholangiocarcinoma	6 (14.0)	3 (12.0)		1 (7.2)	2 (9.5)	
Grading			0.620			0.060
1	15 (34.9)	6 (24.0)		3 (21.4)	1 (4.8)	
2	23 (53.5)	15 (60.0)		11 (78.6)	15 (71.4)	
3	5 (11.6)	4 (16.0)		0 (0.0)	5 (23.8)	
Margin status			0.528			0.635
R0	41 (95.4)	25 (100.0)		13 (92.9)	18 (85.7)	
R1	2 (4.6)	0 (0.0)		1 (7.1)	3 (14.3)	
Number of ELNs^b^	13.4 ± 5.2	17.2 ± 7.6	0.019	18.8 ± 8.1	27.0 ± 13.1	0.030
pN +	13 (30.2)	11 (44.0)	0.146	7 (50.0)	11 (52.4)	1.000

Values in parenthesis are percentages unless indicated otherwise:

*Abbreviations*: *LPD *laparoscopic pancreaticoduodenectomy, *OPD *open pancreaticoduodenectomy, *IQR *interquartile range, *LOS *length of stay, *POPF *postoperative pancreatic fistula, *DGE *delayed gastric emptying, *PPH *postpancreatectomy hemorrhage, *ELNs *examined lymph nodes, *pN* + lymph node positivity

^a^ Values are median (IQR); ^b^ mean ± SD

### Histopathological outcomes for LPD and OPD

An analysis of histopathological outcomes was conducted to further explore the influence of the Heidelberg triangle type on different surgical procedures. The results showed that there was no significant difference in histology, grading, or margin status between type I and type II in patients undergoing LPD or OPD. Importantly, the mean number of ELNs was higher in type II compared to type I in LPD (17.2 ± 7.6 vs. 13.4 ± 5.2, *P* = 0.019) and OPD (27.0 ± 13.1 vs. 18.8 ± 8.1, *P* = 0.030), but there was no significant difference in the rate of lymph node positivity (Table 3).

### Subgroup analysis of type II patients

Subsequently, we conducted a subgroup analysis on the 23 patients each in the type IIa and type IIv categories within type II. The results revealed no significant differences between type IIa and type IIv in terms of the proportion of LPD (56.5% vs. 52.2%, *P* = 0.767), median tumor size (25.0 mm vs. 25.0 mm, *P* = 0.649), and median operation time (400 min vs. 395 min, *P* = 0.991). Notably, the intraoperative blood loss in type IIa was significantly higher than in type IIv (450 mL vs. 200 mL, *P* < 0.001), suggesting that type IIa notably increases the blood loss during PD combined with TRIANGLE operation (Table 4). Furthermore, there were no significant differences between type IIa and type IIv in terms of intraoperative transfusion rates, length of stay (LOS), overall complication rates, POPF (B/C), bile leakage (B/C), chyle leak (B/C), DGE (B/C), PPH (B/C), intra-abdominal infections, and 90-day postoperative mortality (Table 4). Histopathological outcomes showed that there was no significant difference in histology, grading, margin status, mean number of ELNs, or the rate of lymph node positivity between type IIa and type IIv patients (Table 4).

**Table 4:** Subgroup analysis of type 2

Variables	Type IIa (*n* = 23)	Type IIv (*n* = 23)	*P* value
Operative time (min)^a^	400 (316, 452)	395 (330, 460)	0.991
Intraoperative blood loss (mL)^a^	450 (400, 600)	200 (100, 300)	< 0.001
Intraoperative transfusion			0.189
Yes	1 (4.3)	5 (21.7)	
No	22 (95.7)	18 (78.3)	
Surgical type			0.767
LPD	13 (56.5)	12 (52.2)	
OPD	10 (43.5)	11 (47.8)	
Tumor size (mm)^a^	25.0 (20.0, 35.0)	25.0 (20.0, 33.5)	0.649
LOS (days)^a^	14 (11, 21)	14 (12, 22)	0.886
Surgical morbidity	9 (39.1)	11 (47.8)	0.552
POPF (B/C)	5 (21.7)	4 (17.4)	1.000
Bile leakage (B/C)	1 (4.3)	1 (4.3)	1.000
Chyle leak (B/C)	2 (8.7)	1 (4.3)	1.000
DGE (B/C)	3 (13.1)	1 (4.3)	0.601
Intra-abdominal infection	7 (30.4)	8 (34.8)	0.753
PPH (B/C)	2 (8.7)	2 (8.7)	1.000
Reoperation	0 (0.0)	1 (4.3)	1.000
90-day mortality	0 (0.0)	1 (4.3)	1.000
Histology			0.290
Pancreatic head cancer	15 (65.2)	18 (78.3)	
Ampullary carcinoma	6 (26.1)	2 (8.7)	
Distal cholangiocarcinoma	2 (8.7)	3 (13.0)	
Grading			0.881
1	3 (13.1)	4 (17.4)	
2	15 (65.2)	15 (65.2)	
3	5 (21.7)	4 (17.4)	
Margin status			0.232
R0	23 (100.0)	20 (86.9)	
R1	0 (0.0)	3 (13.1)	
Number of ELNs^b^	21.0 ± 11.7	22.1 ± 11.7	0.744
pN +	9 (39.1)	13 (56.5)	0.238

Values in parenthesis are percentages unless indicated otherwise:

*Abbreviations*: *LPD* laparoscopic pancreaticoduodenectomy, *OPD* open pancreaticoduodenectomy, *IQR* interquartile range, *LOS *length of stay, *POPF *postoperative pancreatic fistula, *DGE *delayed gastric emptying, *PPH *postpancreatectomy hemorrhage, *ELNs *examined lymph nodes, *pN* +  lymph node positivity

^a^ Values are median (IQR); ^b^ mean ± SD

### Logistic regression of independent factors for high intraoperative blood loss

We conducted a binary logistic regression to identify factors influencing intraoperative bleeding. To conduct a binary logistic regression, we designated intraoperative blood loss greater than 500 ml (twice the median value) as the predicted outcome, termed “high intraoperative bleeding.” Among the 12 type II patients with intraoperative blood loss surpassing 500 mL, injuries to the named vessels within the triangle accounted for 10 of these cases (83.3%). In the univariate analysis, surgical type (OR, 0.250; CI, 0.087–0.722; *P* = 0.010), Heidelberg triangle classification (OR, 3.000; CI, 1.027–8.762; *P* = 0.045), and tumor size (OR, 5.600; CI, 1.210–25.917; *P* = 0.028) were identified as significant predictors. Subsequent multivariate analysis confirmed the Heidelberg triangle classification (OR, 3.285; CI, 1.031–10.472; *P* = 0.044) and tumor size (OR, 6.521; CI, 1.309–32.498; *P* = 0.028) as independent factors for high intraoperative bleeding (Table 5). Additionally, while the surgical approach’s impact on bleeding was clinically relevant, it did not achieve statistical significance.

**Table 5:** Univariate and multivariate analysis of independent factors for intraoperative blood loss

Variables	Univariate analysis		Multivariate analysis	
	OR(95%CI)	*P*	OR(95%CI)	*P*
Sex, female/male	0.711 (0.256–1.977)	0.513		
Age, > 70/ ≤ 70	1.167 (0.410–3.323)	0.773		
BMI, ≥ 24.0/ < 24.0	0.705 (0.229–2.168)	0.542		
Surgical type, LPD/OPD	0.250 (0.087–0.722)	0.010	0.333 (0.109–1.020)	0.054
Classification of the Heidelberg triangle, type II/type I	3.000 (1.027–8.762)	0.045	3.285 (1.031–10.472)	0.044
Tumor size (mm), > 20/ ≤ 20	5.600 (1.210–25.917)	0.028	6.521 (1.309–32.498)	0.022
ASA score, 2–3/1				
2	3.048 (0.369–25.200)	0.301		
3	1.200 (0.066–21.723)	0.902		
Main pancreatic duct size (mm), > 3/ ≤ 3	0.857 (0.301–2.442)	0.773		
CA19-9 (IU/mL), > 37.0/ ≤ 37.0	1.696 (0.582–4.938)	0.333		
CEA (ng/mL), > 5.0/ ≤ 5.0	2.536 (0.815–7.891)	0.108		
pN + , ±	0.909 (0.321–2.576)	0.858		
Histology, AC, DC/PHC				
AC	0.655 (0.168–2.555)	0.542		
DC	1.528 (0.359–6.501)	0.566		

*Abbreviations*: *BMI* body mass index, *LPD* laparoscopic pancreaticoduodenectomy, *OPD* open pancreaticoduodenectomy, *ASA* American Society of Anesthesiologists, *CA 19–9* carbohydrate antigen 19–9, *CEA* carcinoembryonic antigen, *pN* + lymph node positivity, *AC* ampullary carcinoma, *DC* distal cholangiocarcinoma, *PHC* pancreatic head cancer

## Discussion

Although the TRIANGLE operation may have a survival benefit for patients with pancreatic cancer, its safety has not been adequately studied [25–27]. In this study, we classified the vascular structure of the Heidelberg triangle for the first time and examined the effects of different types on surgical and pathological indexes. Our findings confirm that in PD, type II of the Heidelberg triangle can lead to increased intraoperative blood loss, particularly in LPD, and can increase the number of ELNs. This suggests potential implications for the TRIANGLE operation in LPD, warranting further investigation into its safety and efficacy.

The safety of the TRIANGLE operation remains uncertain, necessitating an exploration of the vascular anatomy within this region. Previous studies on the LGV and DPA have reported that 39.0% of the LGV drains into the PV, while the origin of the DPA is the SPA in 38.5–46.1% of cases, the SMA in 38.5% of cases, and the CHA in 7.7% of cases [13, 28, 29]. The Hiatt classification is commonly used for the classical classification of the hepatic artery [30]. Although Clement and Whitley classifications are widely applied to the celiac trunk, no classification based on the vessels of the Heidelberg triangle has been established [31, 32]. In our study, we observed similar vascular pathways, and the patients with any named vessels crossing the interior of the Heidelberg triangle that impacted the operation were categorized as type II, while the remaining cases were classified as type I. We found that the LGV drained to the PV in 37.0% of the reconstructed patients, and all crossed the interior of the triangle, consistent with previous studies [29]. Moreover, the majority of the arteries crossing the interior of the Heidelberg triangle were identified as replaced right hepatic arteries, corresponding to the Hiatt type 3 classification [30]. Additionally, we observed α-CHA originating from the SMA crossing the interior of the triangle, which corresponds to an absent celiac trunk in the Whitley classification [32] and Hiatt’s type 5 [30]. In addition to the gastroduodenal artery or inferior pancreaticoduodenal artery, the DPA also contributes to the blood supply of the pancreatic head [13]. We found that a small proportion of the DPA originates from the SMA or CHA that crosses the interior of the Heidelberg triangle. Accurate identification of these DPAs prior to surgery is important for the safety of the TRIANGLE operation. We also encountered occasional MCAs of CHA origin crossing the interior of the triangle, similar to the proportion in previous studies [33]. As a result, the presence of named vessels crossing the interior of the Heidelberg triangle was not rare.

Previous studies have shown significant differences in operative time and intraoperative blood loss between the TRIANGLE operation and standard PD [25]. However, there has been a limited analysis of the specific impact of different Heidelberg triangles on the operation. Our study revealed that type II significantly increased intraoperative bleeding compared to type I, potentially due to the presence of the named vessels crossing the interior of the triangle, which posed challenges during the procedure. Previous studies have shown that the presence of a variant right hepatic artery may increase the risk of intraoperative injury, with vascular injuries presenting in about 10% of PD cases and hepatic artery injuries occurring at a rate of 0.5 to 1.7%. Additionally, the LGV injury may contribute to increased intraoperative blood loss [28, 34]. In our study, of the 12 type II patients who experienced intraoperative blood loss exceeding 500 mL, 10 cases (83.3%) involved injuries to named vessels crossing within the triangle. Moreover, hemorrhage from arteries tends to be more challenging to control. Thus, when named arteries, such as the variant right hepatic artery, crossing within the triangle, surgical caution is significant [34]. Our subgroup analysis of type II reaffirmed this, showing that type IIa had significantly greater intraoperative blood loss than type IIv. Therefore, for type II, and particularly for type IIa, careful identification of named vessels within the triangle during surgery is essential to avoid injuring both veins and arteries, enhancing the safety of the TRIANGLE operation. The presence of additional named vessels crossing the interior to the Heidelberg triangle, where lymph nodes tend to be distributed, may explain the higher number of examined lymph nodes in type II than in type I.

Previous studies have indicated that achieving intraoperative hemostasis during LPD is more challenging than in OPD, as bleeding in open surgery can be controlled by direct compression of the pancreatic head, while laparoscopic hemostasis may be less timely and often requires conversion to open surgery [34]. Our study revealed that type II increased intraoperative bleeding in LPD, the median intraoperative blood loss of type II was significantly higher than that of type I (300 mL vs. 200 mL, *P* = 0.030). Conversely, in OPD, the median intraoperative blood loss was comparable between type I and type II. Although the field magnification in laparoscopic surgery usually results in safer dissection and less blood loss, making LPD generally associated with lower intraoperative blood loss compared to OPD, special attention should be paid to the type II Heidelberg triangle when implementing the TRIANGLE operation in LPD. Furthermore, among the patients undergoing LPD combined with the TRIANGLE operation, three were converted to OPD due to named vessel injuries within the type II Heidelberg triangle. This could be attributed to the increased number of blood vessels crossing through the interior of the Heidelberg triangle in type II, leading to a higher risk of bleeding, as well as the inherent difficulty of achieving laparoscopic hemostasis [35].

Previous studies on LPD have demonstrated that it is associated with lower intraoperative blood loss compared to OPD. Additionally, tumor size has been shown to influence intraoperative blood loss; the former benefit can be attributed to the advantages of minimally invasive techniques, while the latter may be due to the richer blood supply in larger tumors [36]. Consistent with these findings, our logistic regression analysis confirmed these trends, although LPD did not reach statistical significance in the multivariate regression. Building upon this, we have, for the first time, identified that the type II Heidelberg triangle is an independent risk factor for significant intraoperative bleeding during PD combined with the TRIANGLE operation. This could be a consequence of an increased number of named vessels within the triangle, leading to injuries during dissection of the Heidelberg triangle. This assertion is supported by the fact that in patients with blood loss exceeding 500 mL, 83.3% experienced this type of injury.

In summary, this study revealed the importance of recognizing the type II Heidelberg triangle in patients undergoing LPD combined with the TRIANGLE operation due to its association with increased intraoperative blood loss. We suggest using detailed preoperative imaging to understand the vascular layout of the Heidelberg triangle, which can aid in surgical planning. When encountering a type IIa Heidelberg triangle, surgeons should possess robust laparoscopic hemostasis skills. Extra caution is imperative during dissection in this region to prevent bleeding. In situations with a higher risk of bleeding, considering an open approach or being prepared to switch during the procedure might be beneficial [37]. Regular training and simulations can further help surgeons handle the challenges related to the type II Heidelberg triangle effectively. Our findings are relevant even for centers without access to thin-section CT (< 1.25 mm) and 3D reconstruction, as preoperative standard contrast-enhanced CT or MRI imaging can still facilitate the acquisition of Heidelberg classification.

This study has some limitations. First, as a retrospective study, the recorded operation time represents the duration of the entire procedure, thereby not demonstrating a significant difference between type I and type II. A prospective study analyzing the time from the start of the operation to specimen excision, known as resection time, would provide a more accurate assessment of the impact of the type II Heidelberg triangle on the procedure. Moreover, this retrospective study has potential selection bias for LPD and OPD, which demands more prospective studies for further exploration.

In conclusion, this study introduces the vascular classification of the Heidelberg triangle for the first time, building upon previous classifications, and establishes the influence of different types on LPD combined with the TRIANGLE operation. By using enhanced CT and reconstructed images, named vessels crossing the interior of the Heidelberg triangle can be identified, enabling surgeons to engage in targeted preoperative planning and enhancing the safety of the TRIANGLE operation in minimally invasive surgery.

## Data Availability

The datasets analyzed during the current study are available from the corresponding author on reasonable request.
